# Geographical assignment of polar bears using multi-element isoscapes

**DOI:** 10.1038/s41598-019-45874-w

**Published:** 2019-06-28

**Authors:** Geoff Koehler, Kevin J. Kardynal, Keith A. Hobson

**Affiliations:** 1 NHRC Stable Isotope Laboratory, Environment and Climate Change Canada, 11 Innovation Blvd, Saskatoon, SK S7N 3H5 Canada; 2 Department of Biology, University of Western Ontario, 1151 Richmond St. N., London, ON N6A 5B7 Canada

**Keywords:** Mass spectrometry, Stable isotope analysis, Element cycles

## Abstract

Wide-ranging apex predators are among the most challenging of all fauna to conserve and manage. This is especially true of the polar bear (*Ursus maritimus*), an iconic predator that is hunted in Canada and threatened by global climate change. We used combinations of stable isotopes (^13^C,^15^N,^2^H,^18^O) in polar bear hair from > 1000 individuals, sampled from across much of the Canadian Arctic and sub-Arctic, to test the ability of stable isotopic profiles to ‘assign’ bears to (1) predefined managed subpopulations, (2) subpopulations defined by similarities in stable isotope values using quadratic discriminant analysis, and (3) spatially explicit, isotopically distinct clusters derived from interpolated (i.e. ‘kriged’) isotopic landscapes, or ‘isoscapes’, using the partitioning around medoids algorithm. A four-isotope solution provided the highest overall assignment accuracies (~80%) to pre-existing management subpopulations with accuracy rates ranging from ~30–99% (median = 64%). Assignment accuracies of bears to hierarchically clustered ecological groups based on isotopes ranged from ~64–99%. Multivariate assignment to isotopic clusters resulted in highest assignment accuracies of 68% (33–77%), 84% (47–96%) and 74% (53–85%) using two, three and four stable isotope groups, respectively. The resulting spatial structure inherent in the multiple stable isotopic compositions of polar bear tissues is a powerful forensic tool that will, in this case, contribute to the conservation and management of this species. Currently, it is unclear what is driving these robust isotopic patterns and future research is needed to evaluate the processes behind the pattern. Nonetheless, our isotopic approach can be further applied to other apex mammalian predators under threat, such as the large felids, providing that isotopic structure occurs throughout their range.

## Introduction

Effective management and conservation of wildlife populations requires an understanding of population structure so that relevant demographic information can be associated with geographic regions^1^. This is particularly true of wide-ranging apex predators that occur at low densities because, in addition to natural processes and habitat loss, these species are often vulnerable to poaching and illegal trade in animal parts. As a result, these factors have contributed significantly to population declines for many large carnivores^2^. For polar bears (*Ursus maritimus*), like any vulnerable but hunted species, determining biologically relevant population boundaries can aid managers in applying sustainable harvest quotas or determine origins of legally and illegally harvested individuals^3^. Unfortunately, due to their highly dispersed distribution and often secretive behaviors, effective tools to delineate population structure of polar bears and other apex predators has proven elusive.

Use of endogenous markers such as genetic fingerprints and naturally occurring stable isotopes in animal tissues have become an important tool in the study of wildlife conservation and management, including enforcement of legislation for both hunted and endangered species^4,5^. Other endogenous markers, such as concentrations of trace elements (i.e. Hg, Cu, Se), similarly do not require more than one capture or sampling per individual and so are well suited to population delineation given the existence of underlying spatial structure in these markers. Analyses of fatty acids have also been used to study dietary intake and are commonly applied to polar bears.^6^ However, the greatest promise to date has been the use of genetic and naturally occurring stable isotopic markers because underlying evolutionary or biogeochemical processes can be extrapolated spatially^7^. Specifically, stable isotopic compositions of inert tissues such as hair and claws are potentially more powerful tracers of origins if they can be linked to trophic interactions and to spatial isotopic gradients. This is because stable isotopes of C, N, S, H, and O vary due to a variety of biogeochemical processes that occur in food webs and thus often have predictable isotopic compositions^8^.

Polar bears have a circumpolar distribution with the majority of the global population (~26,000 individuals)^9^, occurring over vast parts of the Canadian Arctic and sub-Arctic. The species was listed on the United States Endangered Species Act in 2008 and is considered *Threatened* by the Convention on International Trade in Endangered Species (CITES 2008). In Canada, polar bears were placed on Canada’s Species at Risk Act as *Special Concern* in 2011, a designation that was reaffirmed in 2018 because of declining populations and their vulnerability to environmental change. This is because polar bears are highly specialized carnivores, using sea ice as a platform to prey primarily on ringed (*Pusa hispida*) and bearded (*Erignathus barbatus*) seals. They are thus particularly vulnerable to climate change, which is expected to reduce the total extent and duration of ice cover in the Arctic^10–12^. In Canada, the species is divided into 13 subpopulations based on geography and use by indigenous peoples. Since 1976, regulated subsistence and trophy harvest is permitted in some subpopulations and export of polar bear parts are allowed^13^. Therefore, for proper management and conservation of this vulnerable species, it is essential to establish sustainable hunting quotas and ensure that effective enforcement of these quotas occur^3^.

The objective of this study was to determine if values of multiple stable isotopes (^13^C, ^15^N, ^2^H and ^18^O) in adult polar bear hair sampled from known locations could be used to delineate existing subpopulations or to assign polar bears to geographically distinct isotopic groupings for forensic and management purposes. We reasoned that such an apex predator would reflect any underlying marine-based isoscapes in the Canadian Arctic^14^ because top-level carnivores should be good integrators of broad-scale isotopic patterns and variance in foodwebs^15^. In an exploratory manner and without any *a priori* expectation of the nature of marine isoscapes supporting polar bears, we used three groupings of stable isotopes (1) ^13^C and ^15^N, (2) ^13^C, ^15^N, ^2^H, and (3) ^13^C, ^15^N, ^2^H and ^18^O in polar bear hair collected from discrete locations to determine if analysis of added stable isotopes would increase accuracy of assigning bears to subpopulations or isotopic clusters. We realized that continued use of existing management boundaries would be favored, especially if isotopic information could support their use. However, we also allowed the data to reveal naturally occurring subpopulation clusters that presumably reflect the combination of underlying isoscapes and polar bear physiology and feeding ecology. Finally, we created kriged isotopic surfaces to explore existing underlying patterns for each isotope that would provide the basis for future investigations into the mechanisms driving isotopic patterns in this species.

## Results and Discussion

**Figure 1:**
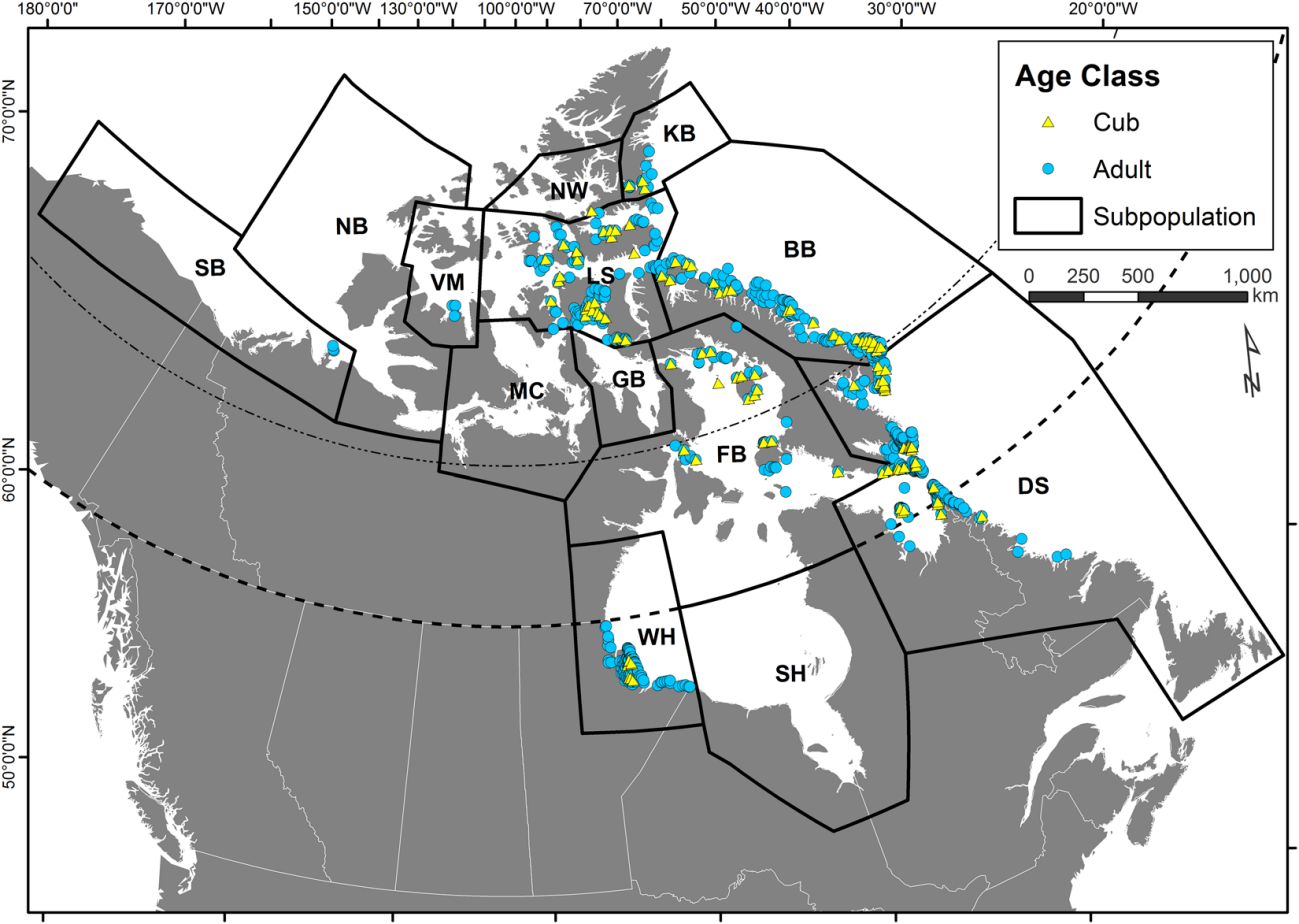
Sampling locations of adult and cub polar bear hair collected for stable isotope (δ^2^H, δ^13^C, δ^15^N, δ^18^O) analysis within polar bear subpopulation boundaries in the Canadian Arctic and sub-Arctic: SB – Southern Beaufort Sea, VM – Viscount Melville Sound, NW - Norwegian Bay, LS – Lancaster Sound, MC – M’Clintock Channel, KB – Kane Basin, GB – Gulf of Boothia, WH – Western Hudson Bay, FB –Foxe Basin, SH – Southern Hudson Bay, BB – Baffin Bay, and DS – Davis Strait.

**Figure 2:**
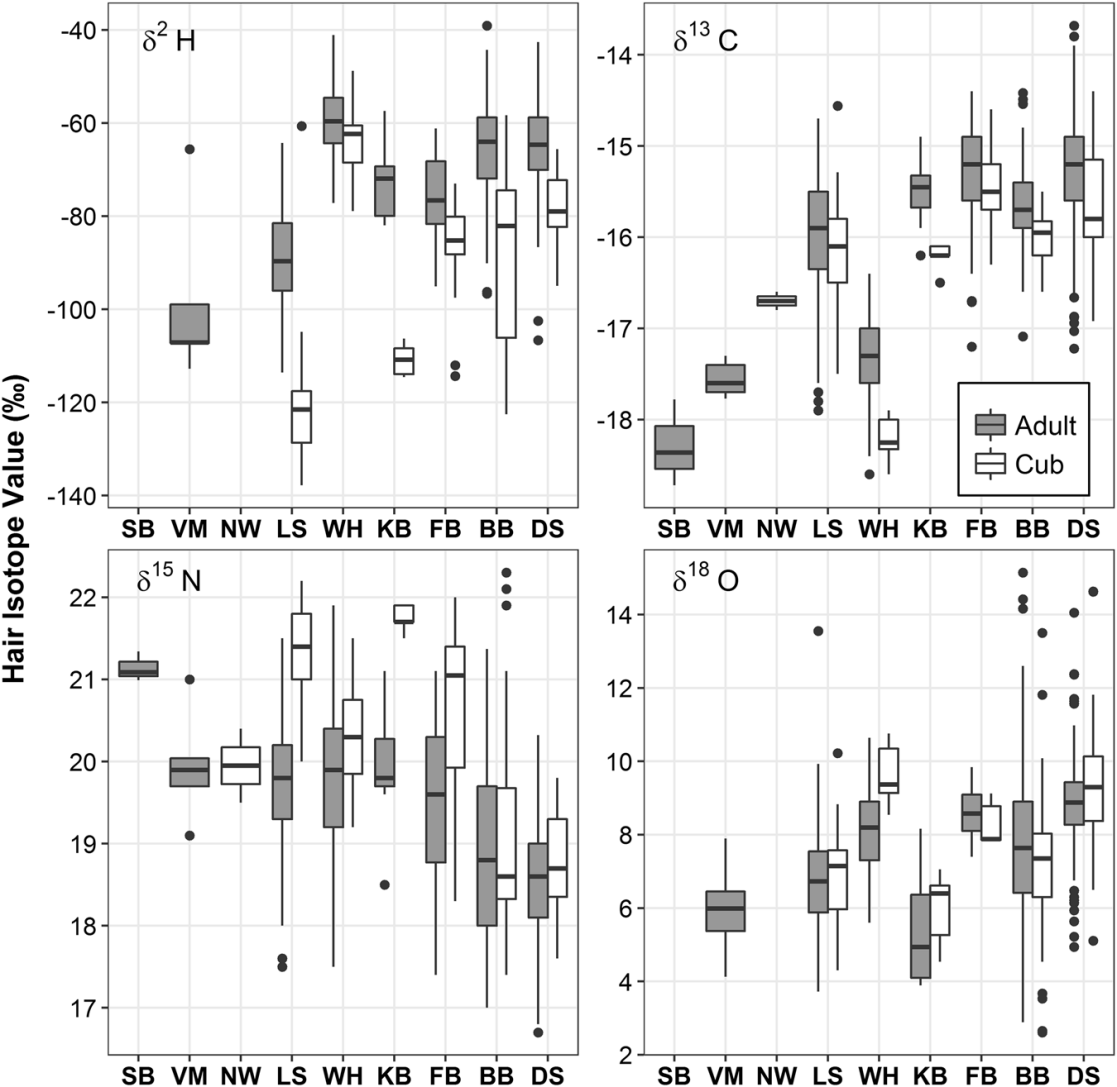
Boxplots showing variation in hair isotope (*δ*^2^H, *δ*^13^C, *δ*^15^N, *δ*^18^O) values of adult (>1 year old) and cub of year (cub; ≤1 year old) polar bears collected within polar bear subpopulation boundaries in the Canadian Arctic and sub-Arctic. See text or Fig. 1 for subpopulation abbreviations. Midlines within boxes indicate the median isotope value, whiskers extend 1.5x beyond the interquartile range, and dots indicate outliers.

We collected samples of polar bear hair from nine of the thirteen subpopulations, which comprise a good part of the Canadian Arctic and sub-Arctic (Fig. 1). The measured *δ*^13^C, *δ*^15^N, *δ*^2^H and *δ*^18^O values of hair from 1,047 adult (>1 yr) and 164 cub (<1 yr) polar bears are shown in Fig. 2. For both adults and cubs we found considerable isotopic variability among subpopulations (Fig. 2); however, it was evident that cubs generally had higher *δ*^15^N and lower *δ*^13^C and *δ*^2^H values than did adults. Using MANOVA on the four stable isotope value matrix (*δ*^13^C, *δ*^15^N, *δ*^2^H, *δ*^18^O), differences in multi-isotope composition between adult and cub polar bears were significant (F_1,917_ = 96.4, *p* < 0.001) when controlling for subpopulation. When contrasting differences in single isotopes between ages controlling for subpopulations using ANOVA, *δ*^2^H (F_1,991_ = 221.6, *p* < 0.001),* δ*^13^C (F_1,1216_ = 52.8, *p* < 0.001) and *δ*^15^N (F_1,1217_ = 81.48, *p* < 0.001) were significant, but not *δ*^18^O (F_1,959_ = 1.2, *p* = 0.28). These isotopic differences between cubs and adults are clearly a result of nursing, which, for polar bears occurs with decreasing frequency up to two years after birth^16^. During nursing, cubs consume maternal milk rich in low *δ*^13^C and *δ*^2^H lipids, which contribute to the body water pool and growth of hair protein. Elevated *δ*^15^N values in cubs can be attributed to trophic effects as is seen in humans and other mammals^17^. For this reason, cubs were not included in further statistical analyses. With adult bears, isotope values varied spatially with *δ*^2^H and *δ*^13^C values generally higher in eastern subpopulations, *δ*^15^N values higher in western subpopulations, and *δ*^18^O being less variable by subpopulation (Fig. 2).

**Figure 3:**
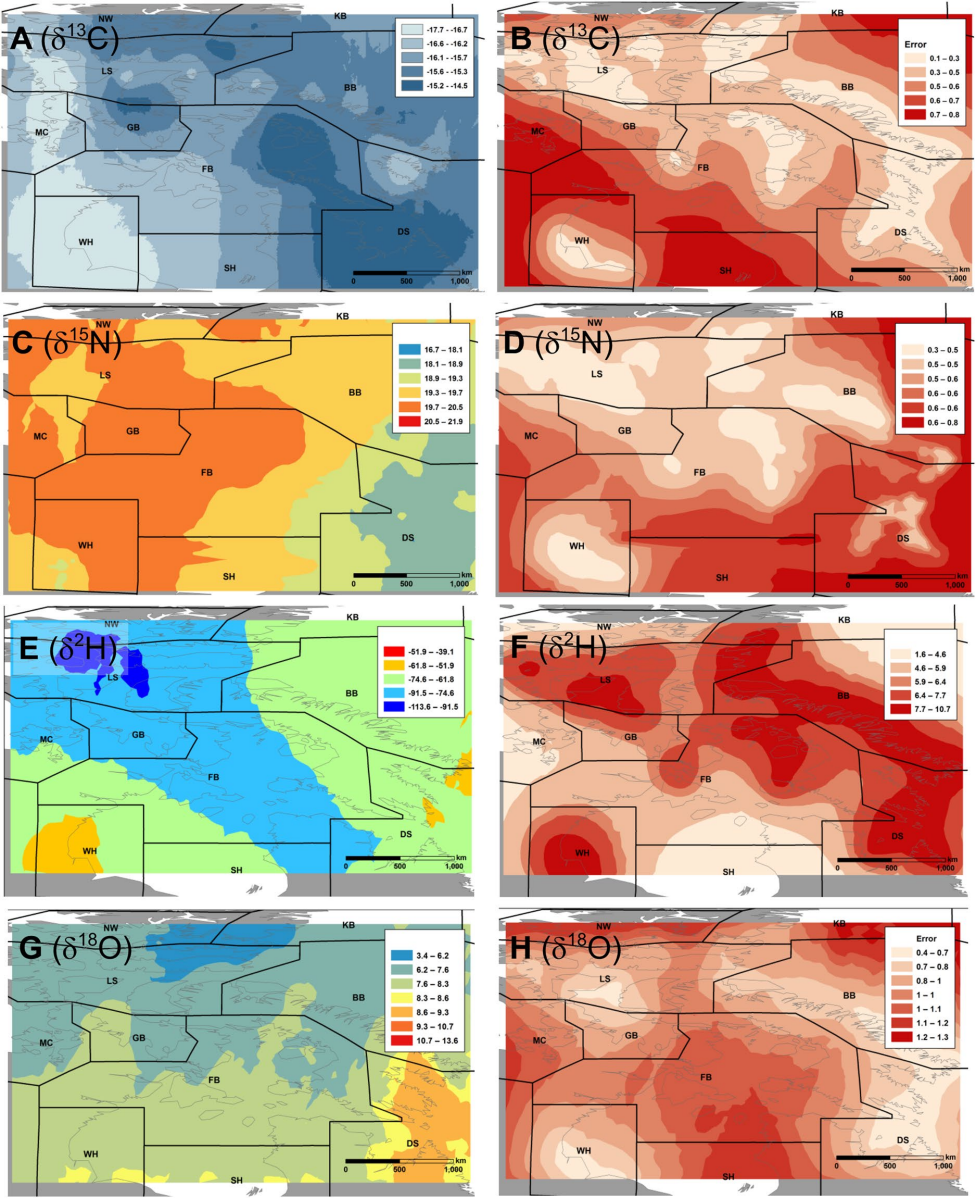
Isoscapes developed using empirical Bayesian kriging for adult polar bear hair samples from the Canadian Arctic and sub-Arctic for four stable isotopes: *δ*^13^C (**A**), *δ*^15^N (**C**), *δ*^2^H (**E**) and *δ*^18^O (**G**). Standard errors in prediction surfaces are paired with each kriged isoscape: *δ*^13^C (**B**), *δ*^15^N (**D**), *δ*^2^H (**F**) and *δ*^18^O (**H**). See Fig. 1 for subpopulation name abbreviations.

The polar bear hair isoscapes interpolated via kriging showed considerable patterns across broad geographic regions of the Canadian Arctic (Fig. 3). Values of *δ*^13^C had substantial longitudinal and latitudinal structure with the lowest values occurring in the area of Western Hudson Bay (WH) and the western portion of Lancaster Sound (LS) and the highest values occurring in the eastern and northern parts of our study area in Davis Strait (DS), eastern Foxe Basin (FB) and parts of LS (Fig. 3A,B). Polar bear hair *δ*^15^N values varied longitudinally with lowest values occurring in the eastern-most regions (Fig. 3C,D). Values of *δ*^2^H had a latitudinal gradient with the WH region having the highest values and LS region having the lowest values (Fig. 3E,F). Values of *δ*^18^O decreased with latitude and were highest in eastern subpopulations, DS and southern Baffin Bay (BB) (Fig. 3G,H).

We assigned bears to existing management subpopulations using CN, CNH, and CNHO isotopic groupings and then assigned bears to grouped subpopulations based on similarities in stable isotope values with hierarchical cluster analysis. We also used polar bear hair stable isotope values to create spatially explicit isotope clusters from interpolated surfaces of individual isotopes and then assigned bears to those groups in a multivariate assignment. We grouped all adult hair samples from across the Arctic and sub-Arctic over a 25-year period, and although most of our samples were collected from 2005–2008, some samples were from other years so it is possible we incorporated some temporal isotopic variability into our models.

We found that adult polar bear hair stable isotope (*δ*^13^C, *δ*^15^N, *δ*^2^H and *δ*^18^O) profiles had considerable spatial structure indicating that environmental isotopic gradients were incorporated into polar bear hair across much of the Canadian Arctic and sub-Arctic. Processes leading to such variation are not well understood or documented but probably reflect a multitude of underlying biogeochemical processes including mixing of isotopically distinct waters, sea-surface temperature differences, carbon sources, and trophic structure^18^. Despite the large geographic sampling region and substantial within- and between-subpopulation isotopic variability, assignment of bears to grouped subpopulations was surprisingly accurate (Table 1), up to almost 100% in some cases. Assignment accuracy varied among subpopulations with Western Hudson Bay (WH) having the highest assignment accuracy and Foxe Basin (FB) having the lowest (99% with three and four isotopes and 0% with two isotopes, respectively). Overall, however, spatial isotopic structure in hair generally did not match the current subpopulation boundaries well. A similar result was found for the geographic variation in genetic structure of the Canadian polar bear population^19^. In contrast to the current management boundaries, this interpolation of isotopic data provides an ecologically-based spatial approach to define subpopulations by identifying geographically explicit, isotopically distinct clusters. Furthermore, these results are informative for forensic and ecological applications in that we can use stable isotopic information to investigate polar bear movements and origins.

**Table 1:** Prediction accuracy of cluster analysis performed using quadratic discriminant analysis using leave-one-out cross validation with various combinations of hair isotope (*δ*^2^H, *δ*^13^C, *δ*^15^N, *δ*^18^O) values collected from adult polar bears in five geographic subpopulations, and three subpopulation groupings in the Canadian Arctic and sub-Arctic (Fig. 1) as the response variable: LS – Lancaster Sound, WH – Western Hudson Bay, FB –Foxe Basin, BB – Baffin Bay, and DS – Davis Strait. Sample sizes (N) without parentheses indicate number of adult bear isotope values in the analyses using *δ*^13^C_h_, *δ*^15^N_h_ and those in parentheses using three or four isotopes.

Subpopulation							Subpopulation Group				
Isotope Cluster	Overall	LS	WH	FB	BB	DS	Overall	FB-LS	WH	BB-DS	N
*δ*^13^C_h_, *δ*^15^N_h_	64.3%	41.3%	96.2%	0.0%	16.8%	89.3%	80.8%	37.9%	95.7%	90.8%	1018
*δ*^13^C_h_, *δ*^15^N_h_, *δ*^2^H_h_	72.3%	78.3%	99.4%	11.1%	45.8%	82.1%	90.2%	63.5%	99.4%	94.2%	819
*δ*^13^C_h_, *δ*^15^N_h_, *δ*^2^H_h_, *δ*^18^O_h_	76.2%	77.1%	99.4%	30.2%	53.1%	84.7%	90.7%	64.0%	99.4%	94.9%	819
N		150 (83)	208 (178)	61 (54)	196 (192)	403 (312)		211 (137)	208 (178)	599 (504)	

**Figure 4:**
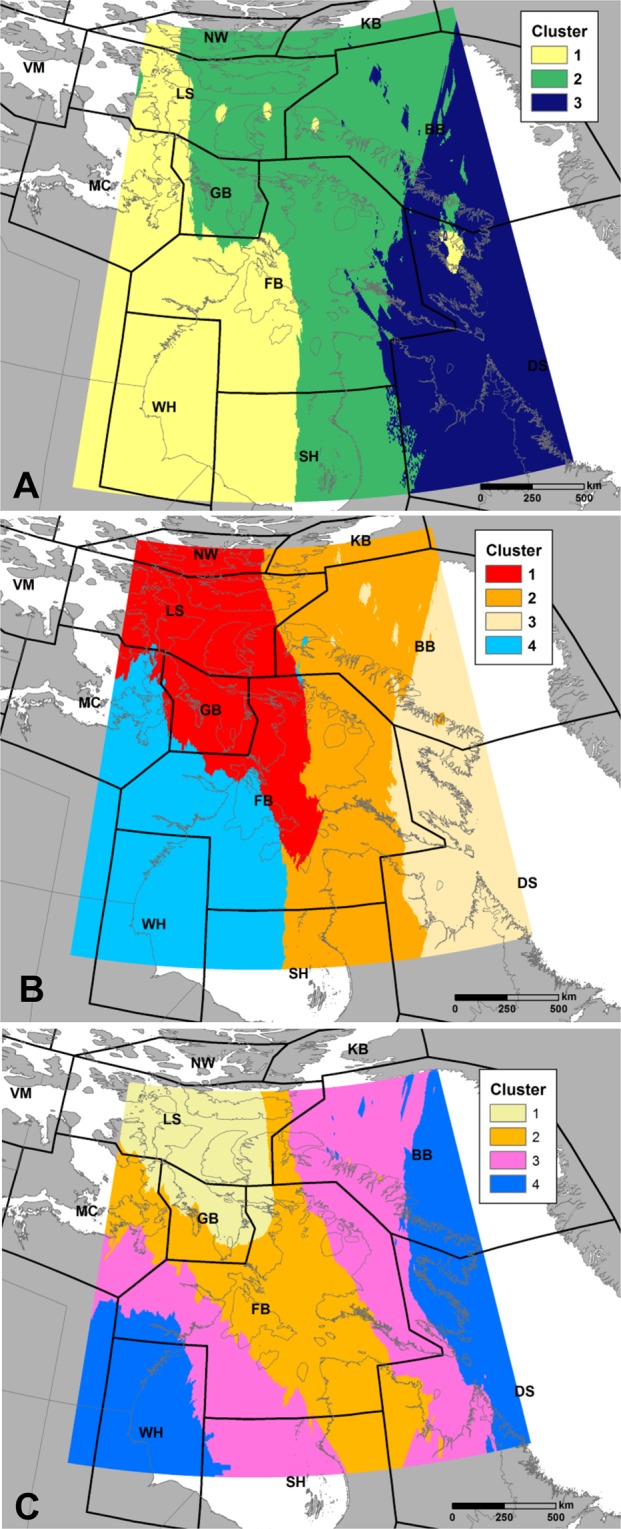
Spatially explicit isotopic clusters derived from polar bear hair isoscapes using ‘clustering for large applications’ (clara) around medoids algorithm for (**A**) *δ*^13^C and *δ*^15^N, (**B**) *δ*^13^C, *δ*^15^N and *δ*^2^H, and (**C**) *δ*^13^C, *δ*^15^N, *δ*^2^H and *δ*^18^O, to which polar bears were assigned using a multivariate cluster method. See Fig. 1 for subpopulation name abbreviations.

A four-isotope (^13^C, ^15^N, ^2^H, ^18^O) approach provided the most accurate solution to assigning bears to subpopulations with the three- and two- isotope combinations consistently providing less accurate solutions (Table 1). Hierarchical clustering of subpopulations resulted in higher assignment accuracies; however, this method relied on grouping individuals from different subpopulations, which substantially lowered the spatial resolution of assignments. Finally, we defined spatially discrete isotopic clusters for polar bear hair that may also be useful for determining origins of bears for forensic or other purposes (Fig. 4). Hierarchical clustering using median values for both the two- (*δ*^13^C, *δ*^15^N) and three-isotope (*δ*^13^C, *δ*^15^N, *δ*^2^H) matrices resulted in three subpopulation groups including: 1) WH, 2) FB-LS, and 3) BB-DS (Table 1). Using this approach, overall correct assignment to isotopic clusters was highest (84%) for the three isotope cluster compared to the two (68%) and four (74%) isotope cluster (Fig. 4). Overall, subpopulations or regions with lower sample sizes typically had lower accuracy rates for assigning individuals and higher error when the isotope data were spatially interpolated.

Using stable isotope measurements to determine origins of polar bears presents some uncertainty owing to multiple factors. Insufficient sample sizes prevented use of our statistical approaches to some of the subpopulations and low sample sizes apparently resulted in lower assignment accuracies for other subpopulations. Polar bear isotope values may vary temporally depending on prey population cycles, food stress and changes in climate, weather or ocean current patterns. Furthermore, polar bears can have large territories and may travel or disperse across or between some subpopulations^20^ thereby integrating isotope values and reducing our ability to assign bears to a specific geographic region, although we tried to reduce this effect by using underfur rather than guard hairs in our analyses. Climatic events, such as the El-Niño Southern Oscillation, may also drive inter-annual variation in some isotopes (e.g. ^2^H and ^18^O) and although the relative coupling of these isotopes from meteoric water into polar bear tissues is unknown, it is possible that this variation may still have an effect.

Despite these potential complications however, it is clear that using these multi-isotope approaches provide a more biologically defensible method to assign polar bears to geographical regions as a result of their integration of broad baseline isotopic patterns across the Arctic. Primarily, isotopic patterns in the diet of polar bears that are expected to vary regionally, will greatly influence bear isotope values and isotopic subpopulation delineation. Relative differences in consumption of various seal species as well as whales and scavenged foods undoubtedly contributes to spatial isotopic variance among bears. However, broader, environmental isotopic gradients driving foodwebs will be manifested over the range of this species and are yet to be fully understood or described. Ultimately, combining other components, such as genetic markers, pollutants, or additional isotopes (i.e. those of Hg, ^34^S or ^37^Cl)^21^ with these stable isotope methods will refine our ability to determine the spatial structure and the underlying ecology of polar bears across these vast regions. At large spatial scales, similar approaches and isotopic tools may be used for management or for forensic applications involving other large predatory species, particularly those under threat from human activities or climate change.

## Methods

### Hair samples

Adult and cub polar bear hair samples were obtained from archived collections held by the Government of Nunavut and from Environment and Climate Change Canada. Samples were collected from across a large portion of the Canadian Arctic and sub-Arctic in nine of 13 subpopulations: Southern Beaufort Sea (SB), Baffin Bay (BB), Davis Straight (DS), Viscount Melville (VM), Lancaster Sound (LS), Norwegian Bay (NW), Kane Basin (KB), Foxe Basin (FB) and Western Hudson Bay (WH; Table 1; Fig. 1). Samples were typically obtained during mark-recapture surveys or from legally harvested bears from 1992 to 2017; however, sampling effort varied among regions and management subpopulations. Adult bears were aged by analysis of the vestigial premolar during the first capture. Bears that were not aged to year were classified as cub of year (COY), yearling, sub-adult or adult. Some bears were sampled more than once in different years including as a cub and as an adult. Hair samples were collected and stored in plastic bags until analysis for stable isotopes.

Polar bear fur was washed in distilled water using an ultrasonic cleaner, dried, and then cut into small fragments using stainless steel scissors prior to cleaning of surface oils in 2:1 chloroform:methanol. Hair is known to vary isotopically in response to changes in diet during growth, an effect that has been demonstrated for polar bears^22^. Underfur was thus selectively sampled to minimize within-year or multiyear stable isotopic differences. We then dried and homogenized hair samples by grinding in a ball mill (Retsch model MM-301, Haan, Germany) to yield approximately 1 g of material.

### Isotope analysis

For carbon and nitrogen stable isotope analyses, we weighed 1 mg of ground hair into precombusted tin capsules. Encapsulated hair was combusted at 1030 °C in a Carlo Erba NA1500 or Eurovector 3000 elemental analyser. The resulting N_2_ and CO_2_ were separated chromatographcally and introduced to an Elementar Isoprime or a Nu Instruments Horizon isotope ratio mass spectrometer. We used two reference materials to normalize the results to VPDB and AIR: BWBIII keratin (*δ*^13^C = −20.18, *δ*^15^N =  + 14.31 per mil, respectively) and PRCgel (*δ*^13^C = −13.64, *δ*^15^N =  + 5.07 per mil, respectively). Within run (n = 5) precisions as determined from both reference and sample duplicate analyses and from QA/QC controls were ± 0.1 per mil for both *δ*^13^C and *δ*^15^N.

For hydrogen and oxygen isotope analyses, we used the method of Hobson and Koehler^23^, which results in hydrogen and oxygen isotope measurements from the same pyrolysis. Briefly, 0.35 mg of ground hair was weighed into silver capsules and thermally converted to H_2_ and CO by reaction with hot glassy carbon at 1400 °C. These gasses were separated chromatographically and introduced by an open split into a Thermo Fisher Delta V isotope ratio mass spectrometer (Bremen, Germany–www.thermofisher.com). To compensate for exchangeable hydrogen, we used the comparative equilibration technique of Hobson and Wassenaar^24^ to measure the nonexchangeable hydrogen stable isotopic compositions. We used Environment Canada keratin reference standards CBS (EC1:Caribou hoof) and KHS (EC2: Kudu horn) to calibrate sample *δ*^2^H (−197 and −54.1 per mil, respectively) and *δ*^18^O values (+2.50 and +21.46 per mil, respectively)^25^. This normalization with calibrated keratins also eliminates any hydrogen isotope measurement bias from production of HCN^26^ in the glassy carbon reactor as described by Soto *et al*.^27^. To minimize any isobaric interferences between the evolved CO and N_2_, the CF capillary was withdrawn during the elution of N_2_^28^. The factory-installed 0.6 m 5 Å mol sieve GC column was replaced with a1.5 m column to further separate these two gasses. Based on replicate (n = 5) within-run measurements of keratin standards and long term analyses of a known QA/QC keratin reference (SPK keratin *δ*^2^H and *δ*^18^O values of −106 and +10.8 per mil, respectively), sample measurement error was estimated at ± 2 per mil for *δ*^2^H and ±0.4 per mil for *δ*^18^O. Although CBS and KHS are not an exact matrix match for hair, Soto *et al*.^27^ have demonstrated that USGS42 and USGS43 hair references can be measured to correct *δ*^2^H values using these reference materials.

### Statistical analysis

Polar bear cubs consume mother’s milk during their first year and any time after during which they are with their mother and thus are expected to have different stable isotope values than adult bears, an effect that has been demonstrated for humans and other mammals^17^. Therefore, we grouped yearling, sub-adult and adults (hereafter ‘adult’) into one group to compare with cub-of-year (hereafter ‘cub’) bears in our analyses. We removed duplicate samples of individual adult bears to avoid potential bias associated with pseudo-replication prior to analysis. We tested for differences in hair stable isotope (*δ*^2^H, *δ*^13^C, *δ*^15^N, *δ*^18^O) values of cubs and adult polar bears and controlled for subpopulation using multivariate analysis of variance (MANOVA) using Pillai’s trace statistic. The F-statistic provided in the MANOVA results is the ‘approximate’ value. We also examined differences between adult and cub hair individual isotope values using ANOVA. We found that cubs generally had higher *δ*^15^N and lower *δ*^13^C and *δ*^2^H values than adults (see Discussion) and based on these results, cubs were excluded from further analyses (Fig. 2). For MANOVA and ANOVA analyses, we removed bears from the SB, VM, NB and KB subpopulations because of low sample sizes (n < 20). For comparisons of cub isotope values, we retained samples from WH, LS, BB, FB and DS subpopulations.

To determine the number of subpopulations that could reliably be distinguished using adult polar bear stable isotope values, we applied hierarchical clustering to our dataset. We then conducted quadratic discriminant analysis (QDA) with leave-one-out cross validation (LOOCV) to the subpopulation groups using the three same isotope combinations as in the previous cluster analysis. The QDA was conducted using the’MASS’ package^29^ in the R computing environment^30^. To this end, we first scaled raw isotope data across subpopulations and produced a dissimilarity matrix with the Euclidean distance measure on median scaled isotope (*δ*^13^C, *δ*^15^N, *δ*^2^H, *δ*^18^O) values for five subpopulations with sufficient data. We contrasted three hierarchical clustering methods (Ward’s, average, complete) to determine which provided the best clustering structure by assessing correlation between the cophenetic distance and original distance matrices. We determined the optimal number of clusters using the ‘silhouette’ method^31^ and identified groups visually by assessing the dendrograms for each hierarchical clustering analysis using the R package ‘fpc’.

Isotope data from five subpopulations (LS, WH, FB, BB, DS) were included in the cluster analyses. We did not have enough samples (n < 20) in each of the remaining subpopulations for meaningful statistical analyses. We created interpolated isotopic landscapes (‘isoscapes’) for adult polar bear *δ*^13^C, *δ*^15^N, *δ*^2^H and *δ*^18^O values from individual bears and used the resulting isoscapes to derive spatially explicit isotopic clusters. Spatial interpolation methods such as kriging are sensitive to outliers and require that data exhibit stationarity; hence, we examined histograms and removed outlier data for each isotope. Adult polar bear hair isotope data was split into training (70%) and test (30%) sets stratified by subpopulation for isoscape development and subsequent analysis. We used empirical Bayes Kriging (EBK)^32^ with k-Bessel detrended semivariograms and a maximum of 500 samples with 500 simulations to create separate interpolated surfaces for adult *δ*^13^C, *δ*^15^N, *δ*^2^H and *δ*^18^O values using the training data sets. EBK is advantageous over other classical kriging methods because it estimates error in the model by accounting for multiple semivariograms versus a single semivariogram. We created the kriged isoscapes in ArcGIS 10.3 (Environmental Systems Research Institute, Redlands, CA.) and used functions in the ‘raster’ package in the R computing environment^33^ to crop and resample kriged isoscapes to ensure matching extents, origins and resolutions.

To determine the number of isotope groupings that provided optimal spatial structure, we merged the four kriged isoscapes into three separate matrices (i.e. raster stacks) with the aforementioned combinations of two, three and four isotopes. We subsequently applied the ‘partitioning around medoids’ (PAM) clustering method on each matrix with a minimum of two and a maximum of 10 clusters using the ‘fpc’ package in R v3.5.0. The resulting optimal number of clusters was verified with the average silhouette width and elbow plot methods. To derive spatially explicit, isotopically distinct clusters we used the ‘clustering for large applications’ (clara) algorithm^34^ on each isotope matrix to assign each cell in the isoscape matrix to a medoid (cluster group). The clara method is similar to PAM but instead of using the entire dataset, it randomly selects a subset of data (i.e. cells in the isoscape matrix) and is thus useful for large datasets. Analyses were conducted with the ‘cluster’ package in R v3.5.0 using Euclidean distance as the dissimilarity measure. To determine the accuracy of assigning polar bears to spatially explicit isotopic clusters based on kriged isoscapes, we used a multivariate assignment technique described in Hobson *et al*.^35^.

Stable isotopic compositions are reported in the delta notation:

1 $$\delta =\frac{{R}_{sample}}{{R}_{std}}-1$$

where R_sample_ is the ratio of the rare (heavy) isotope to the common (light) isotope in the sample, such as ^2^H/H,^18^O/^16^O, etc, and R_std_ is the corresponding absolute ratio of the standard (VSMOW, VPDB, AIR). Delta values are typically close to zero so that for clarity they are multiplied by1000 and are thus expressed in per mil (‰), or parts per thousand.

## Data Availability

The datasets generated during and/or analysed during this study will be available at the ECCC open portal repository, https://open.canada.ca/data/en/dataset?organization=ec or may be obtained from the corresponding author by reasonable request.
